# Self-Assembled Ag-Cu_2_O Nanocomposite Films at Air-Liquid Interfaces for Surface-Enhanced Raman Scattering and Electrochemical Detection of H_2_O_2_

**DOI:** 10.3390/nano8050332

**Published:** 2018-05-15

**Authors:** Li Wang, Huan Qi, Lei Chen, Yantao Sun, Zhuang Li

**Affiliations:** 1 College of Chemistry, Jilin Normal University, Siping 136000, China; 17767963378@163.com (H.Q.); chenlei@jlnu.edu.cn (L.C.); 15504341819@163.com (Y.S.); 2 State Key Laboratory of Electroanalytical Chemistry, Changchun Institute of Applied Chemistry, Chinese Academy of Sciences, Changchun 130022, China; zli@ciac.ac.cn

**Keywords:** nanocomposite films, self-assembly, air–liquid interface, SERS, electrochemistry, sensor

## Abstract

We employ a facile and novel route to synthesize multifunctional Ag-Cu_2_O nanocomposite films through the self-assembly of nanoparticles at an air-liquid interface. In the ethanol-water phase, AgNO_3_ and Cu(NO_3_)_2_ were reduced to Ag-Cu_2_O nanoparticles by NaBH_4_ in the presence of cinnamic acid. The Ag-Cu_2_O nanoparticles were immediately trapped at the air-liquid interface to form two-dimensional nanocomposite films after the reduction reaction was finished. The morphology of the nanocomposite films could be controlled by the systematic regulation of experimental parameters. It was found that the prepared nanocomposite films serving as the substrates exhibited strong surface-enhanced Raman scattering (SERS) activity. 4-aminothiophenol (4-ATP) molecules were used as the test probes to examine the SERS sensitivity of the nanocomposite films. Moreover, the nanocomposite films synthesized by our method showed enhanced electrocatalytic activity towards hydrogen peroxide (H_2_O_2_) and therefore could be utilized to fabricate a non-enzymatic electrochemical H_2_O_2_ sensor.

## 1. Introduction

The facile synthesis of noble metal and metal oxide nanomaterials has attracted considerable attention due to their wide applications in electrochemical catalysis, sensors, energy devices, drug nanocarriers, and surface-enhanced Raman scattering (SERS) detection [1,2,3,4]. Previous studies indicated that noble metals could be coupled into metal oxides to yield diverse metal-metal oxide heterostructures [1], which have been found to exhibit improved properties and functions as novel materials for catalysis [5], gas sensing [6], energy storage [7], electrochemical devices [8,9], and others [10]. Compared with pure metal or metal oxide nanomaterials, the metal-metal oxide nanocomposites showed several obvious advantages. For example, the metal-metal oxide nanocomposites are more stable than their individual components [11]; the introduction of metal oxides into noble metals can extend the light absorption range of the wide band gap of metal oxides [12]; the formation of Schottky barriers in the nanocomposites reduces the recombination of the photo-excited electron holes [13]. In addition, the hybrid system provides high possibility to enhance the optical, electronic, and mechanical properties of materials via the synergistic effects of both components.

Sliver (Ag), a relatively cheap noble metal, has been widely utilized for the synthesis of Ag nanoparticles, nanorods, and nanowires for various applications [14,15,16]. Cu_2_O is a low-cost, environment-friendly *p*-type semiconductor with a narrow band gap (2.17 eV) [17], and therefore Cu_2_O-based materials have been used widely in catalysis, sensors, solar cells, lithium-ion batteries, and water splitting [11,18,19,20,21]. Especially, the combination of Ag with Cu_2_O has attracted strong attentions and a lot of synthesis methods of Ag-Cu_2_O nanocomposites for different applications have been reported [22,23,24,25,26,27,28]. For instance, Yang et al. have reported the controlled preparation of Ag-Cu_2_O nanocorncobs by reacting at 100 °C in an oil bath [24]. It was found that the synthesized Ag-Cu_2_O nanocomposites exhibited enhanced photocatalytic activity under visible light. Xu and co-workers have introduced a graft-immobilization method for the fabrication of Ag-Cu_2_O hybrid nanowires [25]. The conjugation of Ag nanoparticles onto the as-prepared Cu_2_O nanowires was based on the chemical cross-linking of L-dopa. However, the above strategies for preparing Ag-Cu_2_O nanocomposites need long reaction period, high reaction temperature, or complex process, which limit the economic, simple, and large-scale synthesis of Ag-Cu_2_O nanocomposite materials for potential applications. 

Previously, some other methods have been utilized to synthesize Ag-Cu_2_O composite films [29,30,31]. For instance, Pan and co-workers have reported the preparation of Ag-Cu_2_O composite films by electrodepositing Ag nanoparticles onto Cu_2_O films [29]. In another case, Fu et al. have demonstrated the fabrication of Ag-Cu_2_O composite films via the thermal transformation of CuO/Cu(OH)_2_ nanosheets/nanowires templates and subsequent decoration with Ag nanoparticles [31]. In this work, for the first time we demonstrate a simple and novel method for the preparation of multifunctional Ag-Cu_2_O nanocomposite films by using the self-assembly of nanoparticles at the air-liquid interface. Our strategy shows great advantage over other methods in terms of low cost, green synthesis, high stability, and simple operation. The as-prepared Ag-Cu_2_O nanocomposite films as the SERS substrates show striking enhancement. The SERS activity of substrates could be controlled by tuning the morphology and component of the films. In addition, the Ag-Cu_2_O nanocomposite films could be used to fabricate an electrochemical hydrogen peroxide (H_2_O_2_) sensor with the wide linear range of 0.02–3.39 mM and 3.39–11.79 mM, and a low detection limit of 5 μM.

## 2. Materials and Methods

### 2.1. Materials

Cinnamic acid (CA, 99%) was purchased from Alfa Aesar (Shanghai, China). Silver nitrate (AgNO_3_, A. R.), Cupric nitrate (Cu(NO_3_)_2_, A. R.), sodium borohydride (NaBH_4_, A. R.), hydrogen peroxide (H_2_O_2_, A. R.) and ethanol (G. R.) were supplied by Sinopharm Chemical Reagent Co. Ltd. (Shanghai, China). 4-aminothiophenol (4-ATP) was purchased from ACROS (Shanghai, China). All chemicals were used without further purification. The water used throughout the experiments was ultrapure water.

### 2.2. Preparation of the Ag-Cu_2_O Nanocomposite Films

10 mM CA solution was prepared by dissolving CA powder into the mixed solution of ethanol and water (*v*/*v* = 3:7). 1, 3, 6, and 9.6 mL CA solution were diluted to 9.6 mL by water in 40 mL beakers, respectively, then 0.3 mL of 10 mM AgNO_3_ aqueous solution and 0.1 mL of 10 mM Cu(NO_3_)_2_ aqueous solution were added into the above CA solution. After mixing equally, under vigorous stirring, freshly prepared NaBH_4_ aqueous solution (1%, *w*/*v*) was added into the above mixed solution until the color of the colloid changed to filemot, and the Ag-Cu_2_O nanocomposite films appeared simultaneously at the air-liquid interface. The films were transferred onto indium tin oxide (ITO) glass slides with 1.0 cm × 1.0 cm dimension, and dried in the air for further characterization. 

### 2.3. SERS Measurements

The ITO glass slides with the nanocomposite films were immersed in 1.0 × 10^−5^ M 4-ATP ethanol solution for 2 h, and then the films were washed by ethanol and water, and dried in the air for the next SERS measurements. 

### 2.4. Electrochemical Experiments

All electrochemical measurements were performed on a CHI 832D (CH Instruments, Chenhua Co., Shanghai, China) electrochemical workstation with a conventional three-electrode electrochemical system. An Ag/AgCl electrode (saturated KCl), a platinum wire and modified glassy carbon electrode (GCE) (3 mm in diameter) were used as reference, counter, and working electrode, respectively. In the experiments, the Ag-Cu_2_O nanocomposite films were transferred onto the clean GCE, and dried in the air.

### 2.5. Characterizations

Scanning electron microscopy (SEM) images and energy dispersive spectroscopy (EDS) analysis were obtained using an XL30 ESEM FEG field emission scanning electron microscope (FEI Company, Hillsboro, OH, USA) at an accelerating voltage of 20 kV. X-ray photoelectron spectroscopy (XPS) was carried out on an ESCALABMKII spectrometer (VG Co., UK) with AlKα X-ray radiation as the source for excitation. SERS spectra were performed on a Renishaw 2000 model confocal microscopy Raman spectrometer (Renishaw Ltd., Gloucestershire, UK). The 514.5 nm radiation from an air-cooled argon ion laser was used as the exciting source. 

## 3. Results and Discussion

### 3.1. Morphology and XPS Analysis of the Ag-Cu_2_O Nanocomposite Films

**Figure 1:**
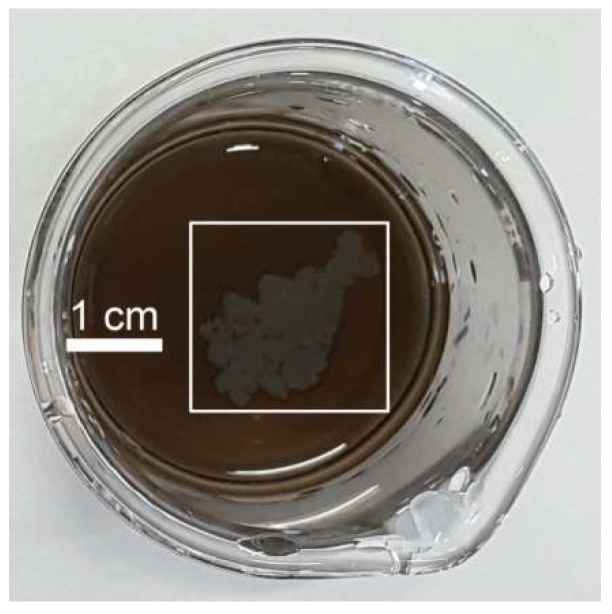
Photograph of the 2D Ag-Cu_2_O nanocomposite film formed at the air-liquid interface (Marked with white rectangle).

**Figure 2:**
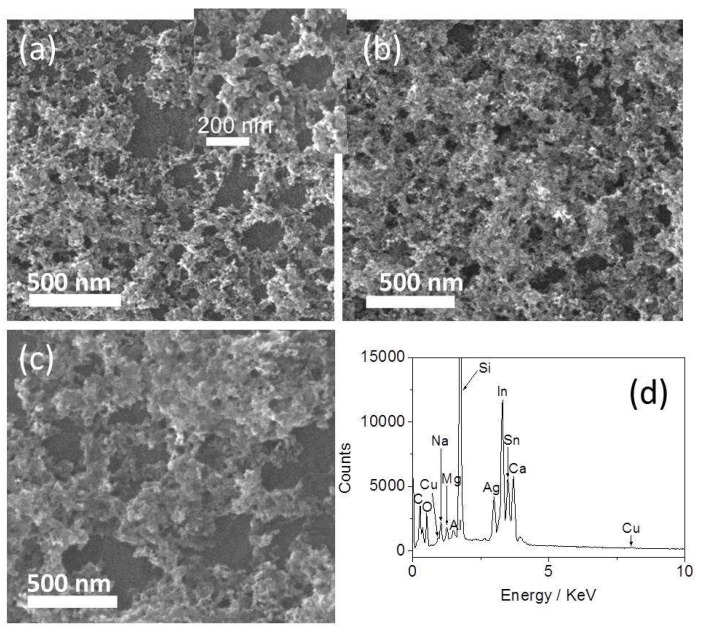
SEM images of the Ag-Cu_2_O nanocomposite films prepared by adjusting the concentration of CA as (**a**) 1 mM; (**b**) 3 mM; (**c**) 9.6 mM; (**d**) EDS analysis of (**b**).

Figure 1 gives a photograph of the two-dimensional (2D) nanocomposite films (marked with the white rectangle) with a surface area of several square centimeters. The morphology of the obtained nanocomposite films were characterized using SEM, and the results are shown in Figure 2. When the concentration of CA in the reaction system was 1 mM, the film was a network structure with aggregated nanoparticles (Figure 2a). The nanoparticles interconnect closely, and there are hardly spaces among the particles (inset of Figure 2a). The size of nanoparticles is uniform with an average diameter of about 37 nm through measuring the diameter of 30 nanoparticles. As the concentration of CA in the reaction system was increased to 3 mM, the film was three-dimensional (3D) aggregates of nanoparticles, and no space among the nanoparticles could be seen (Figure 2b). The interconnected aggregates form a porous nanostructure, which makes the surface of nanocomposite film very rough. Such film has a relatively large surface area. When at a higher CA concentration of 9.6 mM, the film was also composed of 3D nanoparticle aggregates (Figure 2c). Compared with the film shown in Figure 2b, this film is obviously thinner and the size of pores is larger. In this work, CA with a hydrophobic benzene ring covers the nanoparticles surface, which makes the obtained particles hydrophobic. The hydrophobic nanoparticles tend to aggregate and migrate from their bulk colloids toward the interface by the Brown motion, where they are captured by the surface tension of the air-liquid interface. According to our previous report [32], the formation of the nanocomposite films was attributed to the combination of the surface tension and hydrophobic attraction. Figure 2d presents the EDS analysis of the film shown in Figure 2b, which demonstrates that the molar ratio of Ag to Cu in this film is about 7:1 (*n* = 3). It can be seen from Figure 2a–c that the morphology of the nanocomposite films could be controlled through adjusting the concentration of CA in the reaction system.

**Figure 3:**
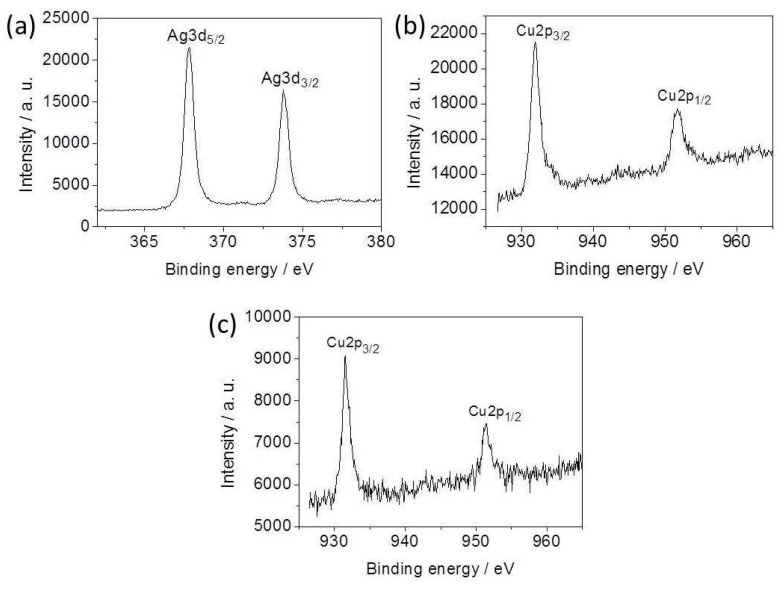
XPS spectra of (**a**) Ag 3d and (**b**) Cu 2p of the as-prepared Ag-Cu_2_O nanocomposite film, and (**c**) Cu 2p of the Cu_2_O nanoparticle film.

XPS was further used to confirm the components of the Ag-Cu_2_O nanocomposite films. Figure 3a,b shows the survey XPS spectrum of the nanocomposite film, calibrated by contaminant carbon (C1s = 284.6 eV). Figure 3a displays the two strong peaks centered at 368.0 and 374.0 eV, belonging to Ag 3d_5/2_ and Ag 3d_3/2_ of Ag^0^, respectively [33,34]. Figure 3b shows the peaks with binding energies of 931.9 and 951.7 eV, corresponding to Cu 2p_3/2_ and Cu 2p_1/2_, respectively, indicating the presence of Cu_2_O in the film [35,36]. The XPS analysis proves that the obtained films are compounds composed of Ag and Cu_2_O. Figure 3c shows the XPS spectrum of pure Cu_2_O nanoparticle film. The binding energies of 931.6 (Cu 2p_3/2_) and 951.4 eV (Cu 2p_1/2_) identify the formation of Cu_2_O nanoparticles. The comparison of Cu 2p peak of the Ag-Cu_2_O nanocomposites with the pure Cu_2_O nanoparticles shows that the binding energy of Cu 2p_3/2_ in the Ag-Cu_2_O composites is higher than that of the pure Cu_2_O nanoparticles, suggesting the interfacial surface charge distribution of the Ag-Cu_2_O nanocomposites and formation of a charge-transfer complex.

### 3.2. SERS Spectra of 4-ATP on the Ag-Cu_2_O Nanocomposite Films

#### 3.2.1. Influence of the Amount of CA in the Reaction System on SERS Activity

**Figure 4:**
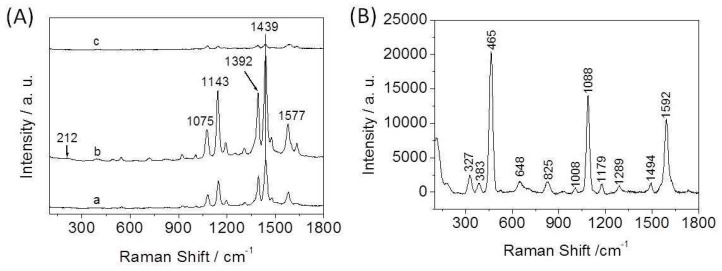
(**A**) SERS spectra of 4-ATP on different Ag-Cu_2_O nanocomposite films obtained by tuning the concentration of CA as (a) 1 mM, (b) 3 mM, and (c) 9.6 mM; (**B**) The normal Raman spectra of solid 4-ATP.

We used 4-ATP as a test probe molecule to evaluate the SERS enhancement ability of the Ag-Cu_2_O nanocomposite films assembled at the air-liquid interface. Figure 4A shows the SERS spectra of 4-ATP on the nanocomposite films. Figure 4B gives the normal Raman spectrum of solid 4-ATP for comparison. The spectrum of solid 4-ATP is similar to that in previous reports [37,38]. Compared with the solid spectrum, the most notable differences in the SERS spectra are frequency shifts and changes in relative intensity for most of the bands. For example, the υ(CS) band at 1088 cm^−1^ in Figure 4B shifts down to 1075 cm^−1^ in Figure 4A, and the υ(CC) band shifts from 1592 cm^−1^ to 1577 cm^−1^. The SERS spectra are similar to those of 4-ATP on Ag surface in previous reports [37,38,39], which reveals that Ag in the nanocomposite films plays a crucial role in SERS enhancement. It should be noted that the nanocomposite films are doped with Cu_2_O, but the SERS enhancement ability of the films is similar to that of the pure Ag nanoparticle films prepared by the same method (data not shown), which implies that Cu_2_O in the films may also plays an additional role in SERS enhancement.

The SERS spectra are dominated by the b_2_ modes at 1143, 1392, 1439, and 1577 cm^−1^. The selective and tremendous enhancement of the b_2_ modes is associated with the charge transfer (CT) mechanism [37,40,41]. CT is highly dependent on the degree of matching between the energy levels of the adsorbed molecules and the metal, and the extent of the charge transfer [42]. For metal oxide, CT depends on the vibronic coupling between the conduction band and valence with the excited state and ground state of molecules [43]. Except for 1075 cm^−1^, the other a_1_ modes are not obviously enhanced. The selective and apparent enhancement of the a_1_ mode suggests that the electromagnetic (EM) field and field gradients also play an important role for the generation of SERS effect [44]. The EM effect may originate from the localized surface plasmon resonances of nanoparticle aggregates in the films. The EM effect is closely related to the geometry of the nanostructured aggregates. We suggest that the factors on SERS enhancements mentioned above can be controlled by adjusting the state of substrates, such as changing the component of substrates, tuning the size and shape of particles, altering the surface roughness of substrate, and so on.

It can be seen from Figure 4A that a SERS signal is observed in each case, but the signal intensity is different. When the concentration of CA in the reaction system is 3 mM, the nanocomposite film as the SERS substrate gives the biggest enhancement compared with the other films. SEM imaging (Figure 2b) shows that this film is composed of 3D aggregates of nanoparticles with many nanoscale pores. The advantages of this morphology are threefold. First, it has relatively large surface area for anchoring many probe molecules, which causes great enhancement. Second, the nanoscale roughness may be favorable for the hot electrons transferring to the probe molecules [45]. The nanoscale roughness with more active sites could make the film have high SERS activity. Third, the nanostructured aggregates could provide a huge EM field to increase the Raman signal. The film shown in Figure 2c gives the weakest Raman signal. With the increase of the concentration of CA in the reaction system, the coverage degree of CA on the particles surface increases. The thick coating is adverse to the adsorption of probe molecules onto the nanoparticles surface. Besides, in this case the amount of ethanol in the reaction system is relatively large, and ethanol is unfavorable to the assembly of nanoparticles at the air-liquid interface [32]. All of these factors cause the activity of this film as the SERS substrate to be weaker than that of the other films. It was found that the signal intensity of the film as the SERS substrate was similar to that of the film as shown in Figure 2b when the concentration of CA was 6 mM. Therefore, we suggest that the obtained nanocomposite films have high SERS activity when the concentration of CA in the reaction system ranges from 3 mM to 6 mM. Based on these results, it can be concluded that the films consisting of 3D nanoparticle aggregates with abundant nanopores serving as the substrates have strong SERS activity. This substrate used for detecting 4-ATP can reach 10^−6^ M, which reveals that the SERS activity of the film is very high.

#### 3.2.2. Influence of the Molar Ratio of Ag to Cu on SERS Activity

**Figure 5:**
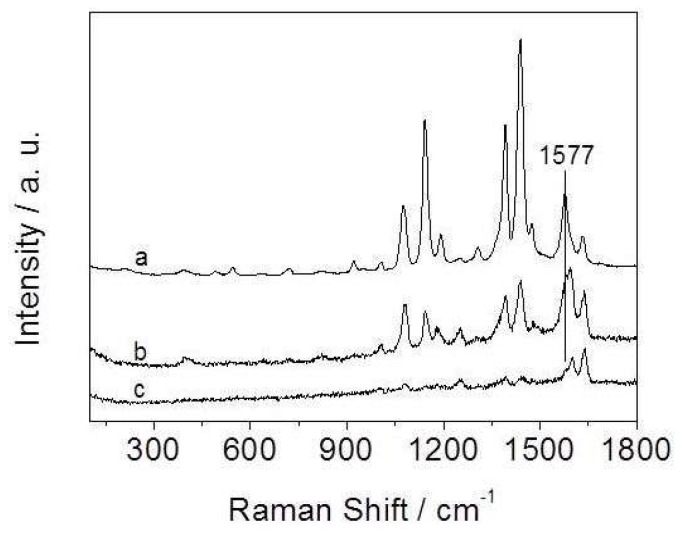
SERS spectra of 4-ATP on the Ag-Cu_2_O nanocomposite films prepared by adjusting the molar ratio of AgNO_3_ to Cu(NO_3_)_2_; (a) 3:1, (b) 1:1, and (c) 1:3.

**Figure 6:**
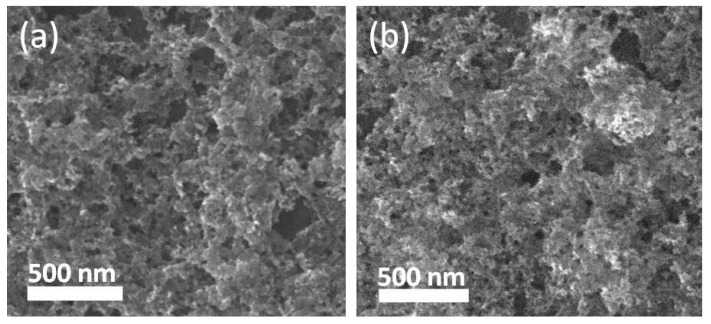
SEM images of the Ag-Cu_2_O nanocomposite films prepared by tuning the molar ratio of AgNO_3_ to Cu(NO_3_)_2_; (**a**) 1:1 and (**b**) 1:3.

In control experiments, it was found that the molar ratio of Ag to Cu had great influence on the SERS activity of the films. Figure 5 shows the SERS spectra of 4-ATP on the nanocomposite films prepared by tuning the molar ratio of AgNO_3_ to Cu(NO_3_)_2_ added into the reaction solution. It can be seen that the SERS signal of the film is strongest when the molar ratio of AgNO_3_ to Cu(NO_3_)_2_ is 3:1 as shown in Figure 5a (The intensity of curve a was reduced by 10 times for easy comparison). The EDS element analysis indicates that the molar ratio of Ag to Cu in this film is about 7:1 (shown in Figure 2d). With the decrease of molar ratio of AgNO_3_ to Cu(NO_3_), the intensity of SERS signal of the films as the substrates gradually decreases from curve a to curve c. SEM images in Figure 6a,b reveal that the morphology of the films is similar to the film shown in Figure 2b. However, the two films have fewer nanopores relative to the film shown in Figure 2b. The EDS element analysis indicates that the molar ratio of Ag to Cu in the films is about 2:1 and 0.45:1 (*n* = 3), corresponding to the films shown in Figure 6a,b, respectively. In other word, the intensity of the SERS signal gradually reduces with the increase of Cu_2_O content in the nanocomposite films. As can be seen from Figure 5, the SERS spectra of the films are considerably different from each other. The frequency of the υ(CC) band at 1577 cm^−1^ obviously shifts to long wavelength with the increase of Cu_2_O content in the nanocomposite films. Also, the relative intensity of the υ(CS) band at 1075 cm^−1^ is different. The apparent changes demonstrate that 4-ATP molecules directly contact the surface of the nanocomposite films to form not only Ag–S bonds but also Cu–S bonds [46]. The difference between Ag–S and Cu–S bonds results in the change of Raman spectra of 4-ATP on different nanocomposite films [47]. The large frequency shift of the b_2_ mode at 1577 cm^−1^ identifies that Cu_2_O participates in CT, as we known that the CT pathway of metal oxides is different from that of noble metal materials. The work function of Ag is ~4.1 eV [48], and the Fermi level of Cu_2_O (~4.84 eV) [49] is larger than that of Ag. Thus, the electrons may migrate from Ag to Cu_2_O for equilibration of the Fermi level between the Ag and Cu_2_O at the interface [48,50,51]. The charge redistribution yields positively charged Ag and negatively charged Cu_2_O with the highest charge density region located adjacent to the junction, which may excite a larger electromagnetic field to enhance SERS intensity [48].

Here it should be noted that the frequencies of the other Raman bands are almost unchanged from curve a to curve b except for the band at 1577 cm^−1^, which suggests that the SERS effect of the nanocomposite films is mainly attributed to the CT from Ag to 4-ATP molecule because Ag is the main component in the two films. When the component of the film is dominated by Cu_2_O (curve c), the intensity of the SERS signal is weaker than that of the other two films. In addition, the band at 1603 cm^−1^ is stronger than the other b_2_ bands, which is totally different from the other curves. This suggests that Cu_2_O in this nanocomposite film may play a main role for SERS enhancement. Meanwhile, Cu_2_O as semiconductors with poor conductivity generally shows much weaker resonance effect, and their SERS activity is much poorer than that of noble metals [36]. Therefore, the SERS activity of the films decreases with the increase of Cu_2_O content in the corresponding film.

### 3.3. Electrocatalysis of the Nanocomposite-Films/GCE towards H_2_O_2_

**Figure 7:**
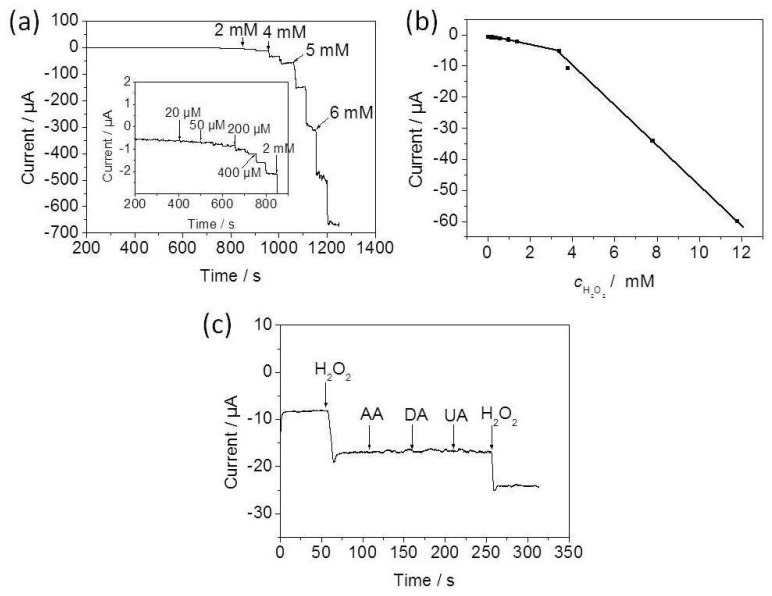
(**a**) Typical *i*-*t* response curve of nanocomposite-film/GCE upon successive additions of different amounts of H_2_O_2_ into 0.1 M PBS at −0.25 V. The inset is the early *i*-*t* response from 200 s to 900 s; (**b**) calibration curve; (**c**) selectivity of the sensor.

In this study, the potential application of the as-prepared Ag-Cu_2_O nanocomposite film for the fabrication of an electrochemical H_2_O_2_ sensor was explored. It was found that the molar ratio of AgNO_3_ to Cu(NO_3_)_2_ of 1:1 promoted the formation of a porous film with uniform structure, and therefore this kind of film was chosen for the fabrication of the electrochemical sensor. Based on the optimal experimental data, −0.25 V was selected as the applied potential for current-time (*i*-*t*) measurement in this work. Figure 7a shows the typical *i*-*t* response of the nanocomposite-films/GCE with successive injection of H_2_O_2_ into 0.1 M PBS (pH 7.4). As H_2_O_2_ was added into the N_2_-saturated PBS solution, a stable current response increased rapidly as a result of reduction of H_2_O_2_ upon continuous additions of H_2_O_2_. The corresponding calibration curve exhibits a regular current response to H_2_O_2_ concentration, and the sensor has two obvious linear detection ranges (Figure 7b). The first linear relation of this sensor for H_2_O_2_ concentration is 0.02–3.39 mM, which can be described by a linear regression equation of I(μA) = −0.514 − 0.00133*c*(μM) (R = −0.9946, *n* = 10). The second linear range is 3.39–11.79 mM, and the linear regression equation is I(μA) = 14.91 − 0.00634*c*(μM) (R = −0.9988, *n* = 4). The detection limit of this sensor is 5 μM at a signal-to-noise of 3. The selectivity of this sensor was tested by comparing the amperometric response for three relevant electroactive species, ascorbic acid (AA), uric acid (UA), and dopamine (DA), respectively, as shown in Figure 7c. It can be observed that the additions of three species do not cause any current response, which demonstrates the high selectivity of the sensor based on the nanocomposite film for the detection of H_2_O_2_.

## 4. Conclusions

In summary, we demonstrated a facile strategy to fabricate multifunctional Ag-Cu_2_O nanocomposite films with good SERS and electrocatalytic activity at an air–liquid interface. The SERS enhancement ability of the films is closely related to the experimental parameters. The results indicate that the as-prepared nanocomposite films are highly efficient SERS substrates on the part to great detection sensitivity, good stability, and reproducibility. Moreover, the electrochemical experiments prove the enhanced electrocatalytic activity of the films towards H_2_O_2_, and the fabricated electrochemical sensor exhibits low detection limit, high selectivity, and long-term stability. We believe that the preparation method of multifunctional Ag-Cu_2_O nanocomposite films shown in this work will be helpful for the design and synthesis of functional nanomaterials for nanodevices, biosensors, and biomedical engineering.
